# Bilingualism and cognitive aging in ethnically diverse older adults: cross-sectional and longitudinal evidence from Health and Aging Brain Study—Health Disparities

**DOI:** 10.1093/geronb/gbag150

**Published:** 2026-07-25

**Authors:** Lihua Xia, Kang Ren, Ina Demetriou, Kshipra Gurunandan, Karin L Meeker, Didac Vidal-Pineiro, Richard Henson, Health and Aging Brain Study (HABS-HD) Team

**Affiliations:** School of Foreign Languages, Huazhong University of Science and Technology, Wuhan, China; Medical Research Council Cognition and Brain Sciences Unit, University of Cambridge, Cambridge, United Kingdom; HUST-GYENNO CNS Intelligent Digital Medicine Technology Center, Huazhong University of Science and Technology, Wuhan, China; GYENNO Science Co., Ltd, Shenzhen, China; Medical Research Council Cognition and Brain Sciences Unit, University of Cambridge, Cambridge, United Kingdom; Medical Research Council Cognition and Brain Sciences Unit, University of Cambridge, Cambridge, United Kingdom; Department of Family Medicine, University of North Texas Health Science Center, Fort Worth, Texas, United States; Department of Pharmacology and Neuroscience, University of North Texas Health Science Center, Fort Worth, Texas, United States; Center for Lifespan Changes in Brain and Cognition (LCBC), University of Oslo, Oslo, Norway; Medical Research Council Cognition and Brain Sciences Unit, University of Cambridge, Cambridge, United Kingdom; Department of Psychiatry, University of Cambridge, Cambridge, United Kingdom; (Psychological Sciences Section)

**Keywords:** Cognitive reserve, Episodic memory, Alzheimer’s disease, Ethnicity, Gender

## Abstract

**Objectives:**

Identifying modifiable factors that shape cognitive aging trajectories is a central goal in aging research. This study examined whether bilingualism is associated with dementia status and longitudinal cognitive decline, and whether associations vary by ethnicity or gender.

**Methods:**

We analyzed Wave 7 data from the Health and Aging Brain Study—Health Disparities (HABS-HD), a longitudinal U.S. cohort of adults aged ≥50 years. Bilingualism was defined by self-reported use of a second language. Cross-sectional analyses included 4,140 participants (1,013 bilinguals); longitudinal analyses included 1,760 participants (522 bilinguals) followed for up to 4 visits at approximately 2-year intervals. Outcomes were baseline dementia status and latent factor scores for executive function and episodic memory derived from 11 neuropsychological tests. Regression and linear mixed-effects models assessed associations, adjusting for age, education, gender, ethnicity, and practice effects.

**Results:**

Bilingualism was not associated with baseline dementia status or conversion to probable dementia. Cross-sectionally, associations varied by ethnicity: Hispanic bilinguals showed higher executive function and episodic memory scores. No advantage was observed in White participants; conversely, Black bilinguals had lower episodic memory scores. Longitudinally, bilingualism was associated with slower episodic memory decline independent of sociodemographic factors; no longitudinal association was observed for executive function.

**Discussion:**

These findings suggest that bilingualism may confer domain-specific benefits for memory trajectories in later life, while cross-sectional differences appear shaped by sociocultural context. The results underscore the importance of considering ethnic diversity and language history when evaluating experiential influences on cognitive aging.

## Graphical Abstract

**Graphical Abstract:**
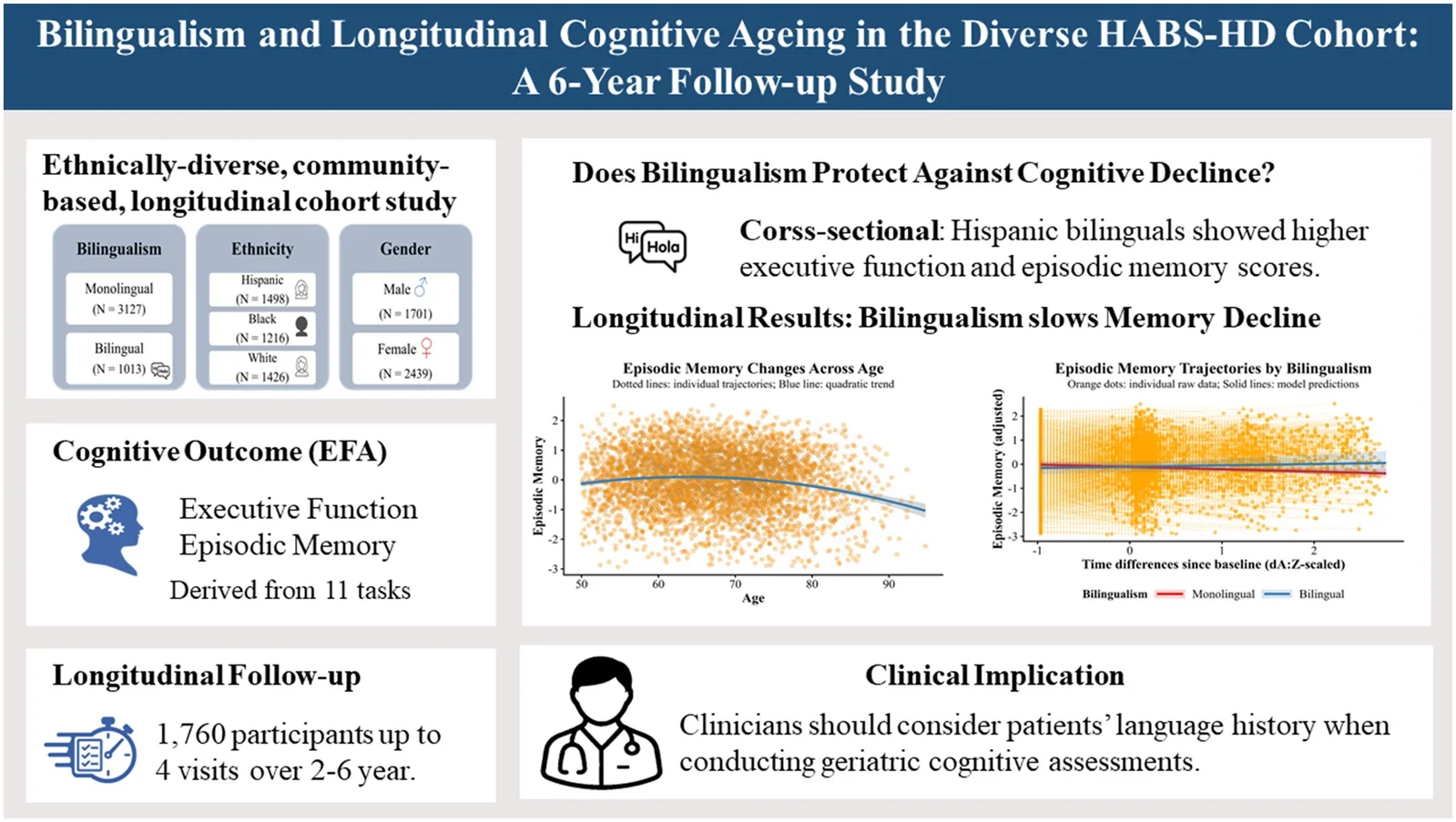

Dementia, from Alzheimer’s disease (AD) or other neurodegenerative diseases, affects millions worldwide and imposes a significant personal, societal, and economic burden. Rates of dementia are projected to rise sharply by 2050, with Hispanic/Latino populations expected to experience the largest increase by 2060 in the United States (Matthews et al., 2019). These trends underscore the importance of early diagnosis and intervention, given that treatments are likely to be most effective in preclinical stages, such as mild cognitive impairment (MCI) (Quiroz et al., 2022). However, delays in diagnosis remain common, particularly among ethno-racially diverse and socioeconomically disadvantaged groups, thereby limiting treatment efficacy and exacerbating disparities in outcomes.

One explanatory framework for individual variability in dementia risk is the concept of cognitive reserve, defined as the capacity to maintain cognitive performance despite the presence of brain pathology associated with disease (Stern, 2012). Classic contributors to reserve include education, exercise, and socioeconomic resources (Duggan & Parikh, 2021). Bilingualism has also emerged as a promising but controversial candidate. Managing two linguistic systems is thought to enhance domain-general executive function, with evidence showing that bilinguals often experience delayed dementia onset by several years relative to monolinguals (Bialystok, 2021). Structural neuroimaging studies further suggest that bilingualism is associated with larger volumes in subcortical and frontal regions (Heim et al., 2019). These findings suggest that promoting bilingual experience earlier in life might be a scalable and culturally sensitive intervention to maintain cognitive health in diverse, older populations. However, the findings remain mixed, with some studies failing to detect such protective effects (Gasquoine, 2016; Zahodne et al., 2014). These discrepancies likely reflect methodological heterogeneity, including limited sample sizes, underrepresentation of diverse populations, reliance on insensitive cognitive measures, and inadequate control for confounds such as education and sociocultural context (Del Maschio et al., 2021). Moreover, most of these studies used cross-sectional data; longitudinal studies provide stronger evidence for causality, but there are fewer such studies, and most have yielded largely null results (Crane et al., 2010; Mukadam et al., 2018; Zahodne et al., 2014). Those protective longitudinal effects that have been found occur only under specific conditions, such as speaking four or more languages (Hack et al., 2019), early-life foreign language instruction (Wilson et al., 2015), or in bilinguals with MCI (Costumero et al., 2020).

The Health and Aging Brain Study–Health Disparities (HABS-HD) provides an opportunity to address these limitations of previous studies. HABS-HD comprises a large, ethno-racially diverse cohort, including Hispanic, non-Hispanic White, and Black older adults, with longitudinal cognitive data. Initial cross-sectional findings from HABS-HD suggest that bilinguals exhibit broadly higher cognitive performance, with a distinct executive function profile (Clark et al., 2024; Grasso et al., 2023; Petersen et al., 2022), but also some sociodemographic advantages (Lee et al., 2025). This aligns with the distinction between passive and active cognitive reserve (Incera & McLennan, 2018; Zahodne et al., 2011). A passive reserve account predicts higher overall cognitive performance, but not necessarily slower cognitive decline. In contrast, an active reserve account predicts that reserve-related experiences will moderate age-related change, leading to slower decline over time. Thus, while higher baseline cognitive function (passive reserve) would lead to fewer older bilinguals falling below conventional cut-offs for dementia, stronger evidence for bilingualism being protective (active reserve) would be slower rates of decline of such cognitive abilities, requiring longitudinal analysis.

HABS-HD data can also be used to test whether any effects of bilingualism are moderated by ethnicity, given that prior research has largely focused on homogeneous ethnic groups. Moreover, most previous studies controlled for gender, but only two small, cross-sectional studies tested whether bilingualism effects are moderated by gender, with mixed findings (Lamar et al., 2019; Subramaniapillai et al., 2019). These omissions are striking because gender and ethnicity strongly shape dementia risk trajectories. For example, Hispanic and Black participants exhibit a higher prevalence of cognitive impairment, cardiometabolic risk factors, and psychosocial stressors compared to Whites, and women often show superior episodic memory despite a higher lifetime risk for AD (Ferretti et al., 2018; Lamar et al., 2019; O’Bryant et al., 2021). These observations underscore the importance of examining how bilingualism interacts with ethnicity and gender to shape cognitive trajectories.

Building on this, the current study represents a cross-sectional and longitudinal investigation of bilingualism within an ethno-racially diverse cohort. Using HABS-HD, we examine cognitive trajectories and dementia outcomes, while systematically exploring interactions between bilingualism, age, gender, and ethnicity. A better understanding of the protective effects of bilingualism as a function of ethnicity and gender may offer potential strategies to mitigate disparities in AD outcomes across historically underserved populations.

## Method

### Data availability

Data came from the Health and Aging Brain Study—Health Disparities (HABS-HD) Wave 7 release (downloaded July 30, 2025): a single-site, longitudinal study at the University of North Texas Health Science Center (UNTHSC) in Fort Worth, Texas. Using community-based participatory research approaches, HABS-HD recruits racially/ethnically diverse older adults (i.e., aged ≥50 years) from the Dallas–Fort Worth area, with an emphasis on underrepresented groups and Alzheimer’s disease related dementia disparities. Note that ethnicity, race, and gender related information was self-reported; see O’Bryant et al. (2021) and Petersen et al. (2025) for more details on enrolment criteria.

Participants completed cognitive and psychosocial assessments at baseline and follow-up visits, scheduled approximately every 2 years (the interval between Waves 2 and 3 was ∼2.6 years due to COVID-19). The protocol was approved by the UNTHSC Institutional Review Board, with exempt approval for secondary analysis from the University of Texas at Austin (STUDY00003075).

### Bilingualism status

Consistent with prior HABS-HD studies, participants answering “Yes” to “Do you speak a secondary language?” were classified as bilingual; others were classified as monolingual (Clark et al., 2024; Petersen et al., 2025). The protocol does not distinguish between multilingual speakers; thus, “bilingual” refers to speaking at least one additional language. Testing was conducted in English or Spanish according to participant preference. Because HABS-HD did not include detailed measures of current language dominance, English immersion history, or daily language use, first language was used only as the available language-history measure and was not treated as equivalent to dominant language. This may introduce heterogeneity within first-language groups and could attenuate associations involving L1 proficiency; therefore, L1 proficiency analyses were treated as supplementary and interpreted cautiously. In these supplementary analyses, lower first-language (L1) proficiency in bilingual Hispanics (Supplementary Section 1.3) suggests L1 skills did not drive cognitive advantages (see Results). While bilingualism is ideally examined as a continuum, detailed L2-history measures in HABS-HD, including L2 proficiency, switching frequency, and age of acquisition, had substantial missingness (>90%). Therefore, the primary analyses used the binary bilingualism measure, while exploratory analyses on available L2-history measures suggested that L2 proficiency was the primary factor supporting cognitive function (Supplementary Section 1.4).

### Cognitive measures

Participants completed a comprehensive cognitive battery consisting of (a) general cognitive and functional abilities as measured by the Clinical Dementia Rating (CDR) scale and the Mini-Mental Status Examination (MMSE), (b) attention/executive function as measured by the Trail Making Test Parts A and B (TMT-A and TMT-B), Digit Span Test (forward and backward; DSF and DSD), Digit Symbol Substitution Test (DSST), (c) verbal memory as measured by Logical Memory I and II (LM I and II), and Total Learning and Delayed Recall from the Spanish English Verbal Learning Test (SEVLT-total and SEVLT-DR), (d) language fluency as measured by the Animal Naming (semantic fluency) and FAS (phonemic fluency), and (e) language competence as measured by the American National Adult Reading Test (AMNART; English speakers), and Word Accentuation Test (WAT; Spanish speakers) (for detailed information about the tasks, see Petersen et al. [2025]). Time-based scores were reverse-coded (i.e., reversing the sign) to ensure that higher values indicated better performance across all scores.

### Dementia status

Dementia status in the HABS-HD cohort has been determined as cognitively unimpaired (CU), MCI, or Dementia, using a standardized algorithmic decision tree, followed by adjudication by an interdisciplinary clinical consensus panel. An informant interview was conducted to complete the CDR scale, administered by clinicians with expertise in dementia assessment, and both CDR scores and cognitive test performance were considered in making the diagnosis. For further details, see O’Bryant et al. (2021) and Petersen et al. (2025). However, for the present analysis, we combined the MCI and Dementia groups into a single probable dementia (PD) group, given that there were so few Dementia cases in some of the subgroups used for analyses (see Results later)

### Statistical analysis

All analyses were conducted in R Version 4.4.2 (R Foundation for Statistical Computing). Two primary outcomes were examined: (a) binary dementia status (CU vs. PD) and (b) continuous cognitive abilities (composite factor scores). Analyses were performed at both baseline (cross-sectional) and across follow-up visits (longitudinal).

### Baseline (cross-sectional) analyses

Dementia status (PD = 1, CU = 0) was analyzed using logistic regression, with bilingualism, ethnicity, and gender as predictors and baseline age (second-order polynomial) and years of education as z-scored covariates. Models included main effects and all interactions among bilingualism, age, gender, and ethnicity; inference focused on effects involving bilingualism. Z-scored cognitive outcomes were analyzed using linear regression with the same predictor structure. Multiple comparisons were controlled for using the Holm–Bonferroni correction across the bilingualism-related effects of interest. Overall effects were evaluated using type-II sum-of-squares, and effect sizes were reported using odds ratios (OR) or partial eta-squared (*η*^2^*p*).

To derive composite measures of cognitive abilities, we employed a two-step data reduction procedure. First, all 11 cognitive test scores were z-scored to ensure comparability across different measurement scales. Second, a principal component analysis (PCA) was conducted to identify the optimal number of latent cognitive dimensions underlying the observed measures. PCA was performed across all participants and data points to maximize sample size (see Supplementary Section 1.2 and Supplementary Table S14 for longitudinal factor analysis and confirmation of measurement invariance). The number of components was estimated with parallel analysis using *fa.parallel*() from the psych package. PCA was chosen instead of single cognitive test scores in order to capture shared variance that reflects broader cognitive domains rather than task-specific variance (given the correlation between the test scores), as well as to reduce measurement noise and the multiple comparison problem. Based on PCA results, we performed an exploratory factor analysis (EFA) with *oblimin* rotation to allow for correlated factors, which better reflects the theoretical interdependence of cognitive domains. Factor scores were extracted from the EFA solution and served as the dependent variables in the above regression analyses.

### Longitudinal analyses

Longitudinal analyses included participants with at least two visits (range: 2–4). Analysis of dementia conversion was restricted to participants classified as cognitively unimpaired at baseline and modeled using logistic regression. Due to model non-convergence and zero conversion events among Black bilinguals, this subgroup was excluded, and the model was restricted to primary two-way interactions between bilingualism and age, gender, or ethnicity.

Longitudinal cognitive change was examined using linear mixed-effects models with participant-level random intercepts. Fixed effects included bilingualism, ethnicity, gender, education, baseline age (A0; capturing cross-sectional effects), time since baseline (dA) for each timepoint (capturing longitudinal change), and practice effects (modeled as a factor). The primary effects of interest were interactions between bilingualism and dA: (a) bilingualism × dA, (b) bilingualism × dA × ethnicity, (c) bilingualism × dA × gender, and (d) bilingualism × dA × gender × ethnicity. Effects of bilingualism without dA were not reported, as they largely overlap with cross-sectional results.

### Transparency and openness

We report how we determined our sample size and describe all data exclusions, manipulations, and measures in the study. All data, analysis code, and research materials are available via OSF: https://osf.io/tfmy9/overview. This study’s design and its analysis were not preregistered.

## Results

### Baseline (cross-sectional) analysis

#### Overall monolingual and bilingual sample characteristics

The baseline dataset included 4,140 participants (monolinguals: 3,127; bilinguals: 1,013), with demographic characteristics in Table 1. Ethnic composition differed markedly: bilingual participants were predominantly Hispanic (82.6%), whereas monolingual participants were more likely to be White (41.0%) or Black (37.8%) (χ^2^(2,4140) = 1271.80, *p* < .001). Across ethnic groups, bilingual and monolingual participants were largely comparable in age MMSE scores. Among Hispanic participants, bilinguals had significantly higher education levels, χ^2^(1) = 255.91, *p* < .001, and MMSE scores, χ^2^(1) = 212.73, *p* < .001, and were more likely to be female, χ^2^(1) = 6.85, *p* = .009. For Black participants, group differences were non-significant for age, education, and MMSE, though bilinguals were more likely to be male, χ^2^(1) = 12.72, *p* < .001. Among White participants, bilinguals also showed higher education levels, χ^2^(1) = 27.80, *p <* .001, while age, MMSE, and gender composition did not differ significantly. SASH scores revealed distinct differences in language-use patterns across ethnic groups: Hispanic participants reported roughly equal use of Spanish and English in daily life (*M *= 2.90, *SD *= 1.33), whereas Black and White participants reported predominantly English use (*M *= 4.79, *SD *= 0.39 and *M *= 4.80, *SD *= 0.51, respectively).

**Table 1:** Basic demographic characteristics of participants across language groups.

Variable	Hispanic		Black		White	
	Monolingual (*n *= 661)	Bilingual (*n *= 837)	Monolingual (*n *= 1,183)	Bilingual (*n *= 33)	Monolingual (*n *= 1,283)	Bilingual (*n *= 143)
Age, *M* (*SD*)	63.16 (7.99)	63.16 (8.18)	63.29 (7.85)	61.42 (6.47)	68.69 (8.74)	68.57 (9.08)
**Gender, *n* (%)**						
Male	202 (30.6)	311 (37.2)	444 (37.5)	23 (69.7)	545 (42.5)	68 (47.6)
Female	459 (69.4)	526 (62.8)*	739 (62.5)	10 (30.3)*	738 (57.5)	75 (52.4)
Education, *M* (*SD*)	8.17 (4.60)	12.05 (3.88)*	14.62 (2.64)	15.42 (2.32)	15.43 (2.58)	16.57 (2.36)*
MMSE, *M* (*SD*)	24.52 (4.34)	27.28 (2.68)*	27.54 (2.65)	27.42 (2.22)	28.60 (2.25)	28.80 (1.72)
**Dementia status, *n* (%)**						
Unlabeled	4 (0.6)	2 (0.2)	9 (0.8)	0 (0.0)	1 (0.1)	0 (0.0)
Probable dementia	209 (31.6)	204 (24.4)	393 (33.2)	18 (54.5)	266 (20.7)	21 (14.7)
Cognitively unimpaired	448 (67.8)	631 (75.4)	781 (66.0)	15 (45.5)	1,016 (79.2)	122 (85.3)
SASH score, *M* (*SD*)	1.47 (1.29)	2.90 (1.33)	5.00 (–)	4.79 (0.39)	5.00 (0.00)	4.80 (0.51)

*Note*. MMSE = Mini-Mental State Examination. Short Acculturation Scale for Hispanics (SASH) was rated on a 5-point scale (1 = only Spanish, 2 = Spanish better than English, 3 = both equally, 4 = English better than Spanish, 5 = only English). A dash (–) indicates not applicable. For SASH, missingness was 27.5% in Hispanic monolinguals, 12.8% in Hispanic bilinguals, 43.3% in White monolinguals, 18.9% in White bilinguals, 99.9% in Black monolinguals, and 39.4% in Black bilinguals.

* Indicates bilingual group difference, *p* < .05.

#### Dementia status as outcome

Using the HABS-HD definition of dementia (see *Method*), logistic regression indicated that a higher probability of dementia was associated with older age (χ^2^(2) = 64.99, *p* < .001, *η*^2^*p* = .009) and fewer years of education (χ^2^(1) = 32.62, *p* < .001, *η*^2^*p*=.010), as expected. Gender was also a significant predictor, χ^2^(1) = 70.56, *p* < .001, *η*^2^*p* = .02, though interestingly, female participants had a lower probability of dementia (OR = 0.83, 95% CI [0.73, 0.94]). There was an effect of ethnicity, χ^2^(2) = 93.35, *p* < .001, with White participants showing a lower probability of dementia than Hispanic participants (OR = 0.67, 95% CI [0.48, 0.93]), but Black participants showed a higher probability than Hispanic participants (OR = 5.04, 95% CI [2.21, 20.50]). However, once controlling for these factors, no effects involving Bilingualism reached our corrected significance level (see Table S1).

One problem with the HABS-HD data is that bilingualism is imbalanced across ethnicities, i.e., the bilingualism and ethnicity predictors are correlated, such that including both in the same statistical model (as here) might reduce the chance of detecting a true effect of either, or at least attenuate the effect size(s). We therefore repeated the analyses on the Hispanic group alone as it had the most balanced proportion of monolinguals and bilinguals. This showed that bilingual participants showed a lower probability of probable dementia after adjustment for age, gender, and education (OR = 0.84, 95% CI [0.74, 0.96]; see Table S2).

#### Cognitive performance

The results of the PCA analysis are shown in Figure S1. Parallel analysis on resampled data suggested that two components were sufficient, explaining 66.1% of the data variance. The EFA with two factors and oblique rotation resulted in a factor that we labelled Executive Function (capturing 35.1% of the variance), since it loaded significantly on the TMT-A, TMT-B, DSST, Animal fluency, FAS, SEVLT-total, DSF and DFD tasks, and a second factor that we labelled Episodic Memory (capturing 24.2% of the variance), since it loaded on LM I, LM II, SEVLT-total, and SEVLT-DR tasks (see Table S3 for precise loadings across tasks, also see Table S4 for the four-wave configural longitudinal structural equation modelling framework).

**Figure 1:**
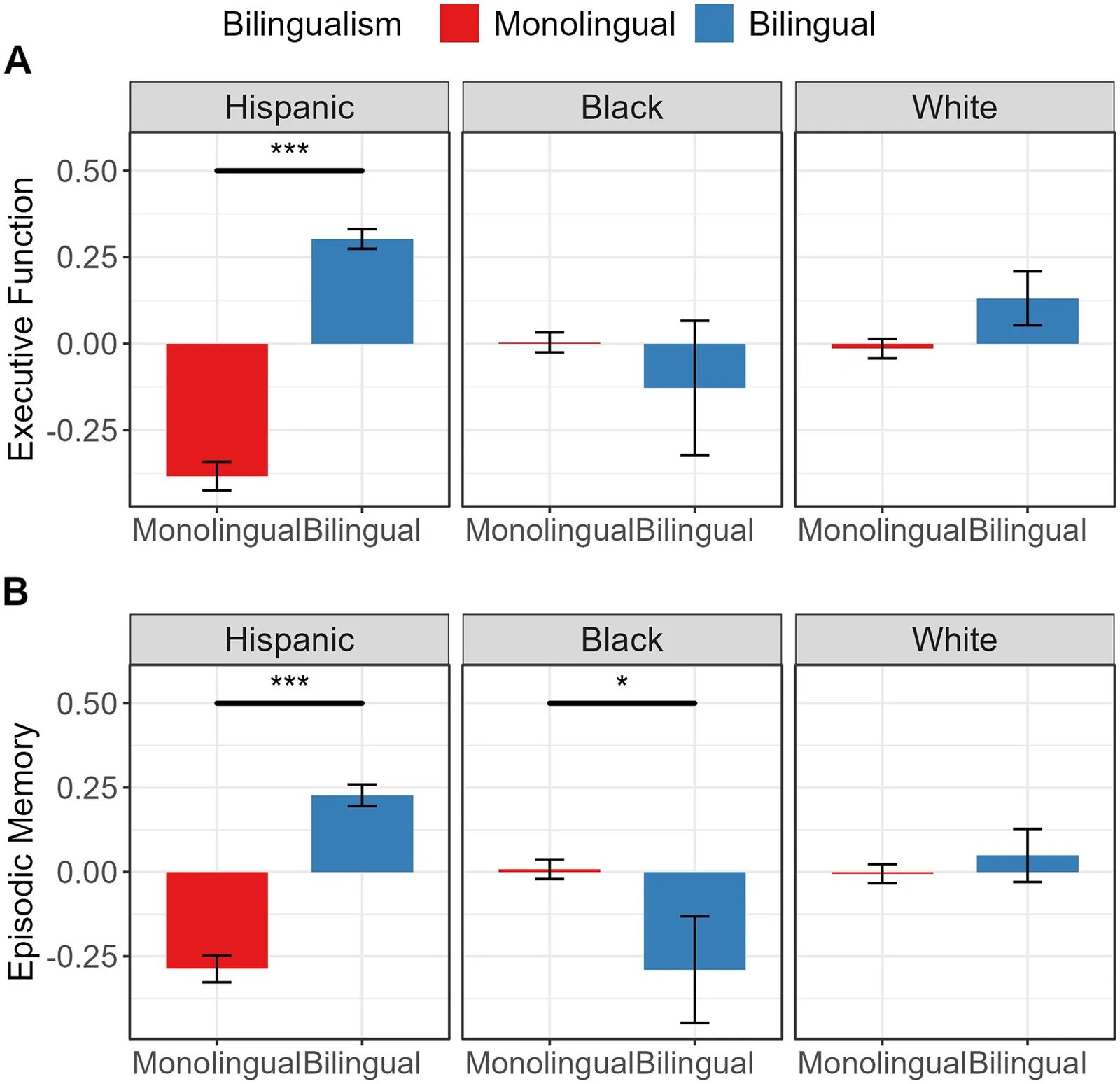
Baseline *z*-scores for executive function (A) and episodic memory (B) split by bilingualism and ethnicity. Error bars show ±1 *SE*. ***p < .001. *p < .05.

Multiple regression showed that executive function decreased with age, *F* (2, 4103) = 483.93, *p* < .001, *η*^2^*p* = .19, and increased with education, *F* (1, 4103) = 1291.76, *p* < .001, *η*^2^*p* = .24, as expected. Female participants also outperformed male participants, *F* (1, 4103) = 134.94, *p* < .001, *η*^2^*p*=.03. A significant main effect of ethnicity emerged, *F* (2, 4103) = 165.61, *p* < .001, *η*^2^*p* = .07, with White participants scoring highest and Black participants lowest (see Table S5). Importantly, there was also a two-way interaction between bilingualism and ethnicity, *F* (2, 4103) = 11.74, *p* < .001, *η*^2^*p<* .001. Follow-up analyses suggested a bilingual advantage in Hispanic participants, β = 0.10, 95% CI [0.07, 0.14], but not in Black or White participants (Figure 1A and Table S6).

Similar to executive function, episodic memory scores decreased with age, *F*(2,4103) = 125.30, *p* < .001, *η*^2^*p =* .06, increased with education, *F*(1,4103) = 502.11, *p* < .001, *η*^2^*p =* .11, were higher in female participants, *F*(1,4103) = 156.09, *p* < .001, *η*^2^*p =* .02, and showed the same ordering across ethnicities, *F*(2,4103) = 169.07, *p* < .001, *η*^2^*p =* .11 (see Table S5). Again, the effects of bilingualism varied by ethnicity, *F* (2, 4103) = 5.61, *p* = .03, *η*^2^*p =* .003, with a bilingual advantage among Hispanic participants, β = 0.11, 95% CI [0.06, 0.16], but a bilingual disadvantage among Black participants, β = −0.18, 95% CI [−0.34, −0.03] (Figure 1B and Table S7-S8).

#### Longitudinal analysis

In the longitudinal sample, 522 participants were bilinguals and 1,238 monolinguals, and their demographics are presented in Table S13.

#### Dementia progression

The probability of converting from a cognitively unimpaired status to probable dementia increased with age, χ^2^(2) = 45.99, *p* < .001, *η*^2^*p =* .04, and decreased with education, χ^2^(1) = 3.99, *p* = .046, *η*^2^*p <* .001. Female participants also had a lower risk than male participants OR = 0.70, 95% CI [0.59, 0.83], while Hispanic participants had a higher risk than White participants OR* = *0.56, 95% CI [0.41, 0.75] (see Table S15). The number of Black participants with available follow-up data was insufficient for reliable analyses (see *Method*). However, no effects of bilingualism reached significance.

#### Cognitive performance

The mixed-effects model on Executive Function revealed an interaction between baseline age (A0) and change in age (dA), indicative of accelerating decline, χ^2^(1) = 106.00, *p* < .001, *η*^2^*p =* .04. No other effects involving dA were significant, including any interactions with bilingualism (Table S16).

**Figure 2:**
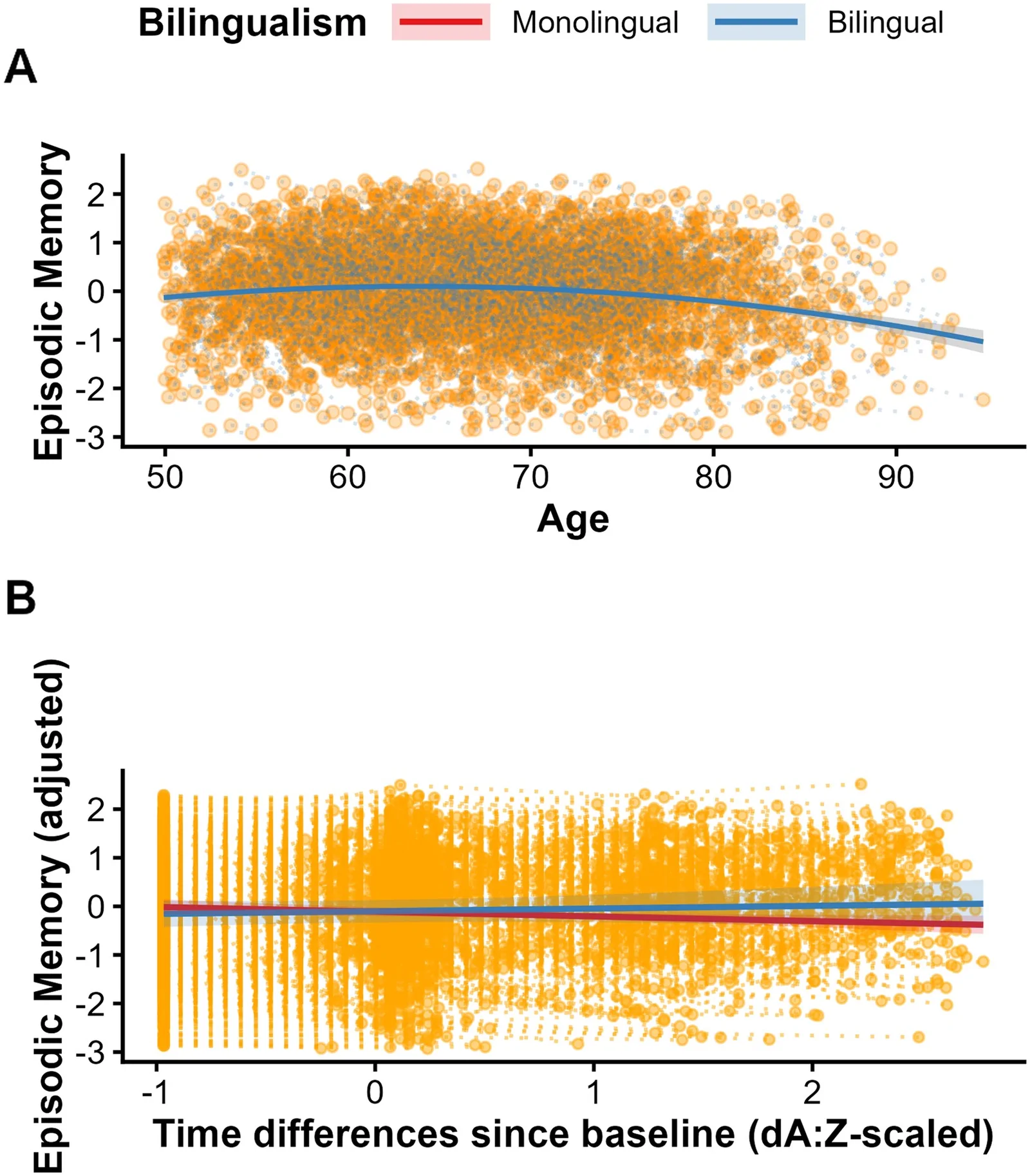
Longitudinal trajectories of episodic memory. (A) Individual episodic memory scores across age (collapsed across ethnicity, gender, and bilingualism) to illustrate a general trend of decline that accelerates with baseline age. Each participant contributed two to four observations (dots) connected by dotted lines to illustrate within-person trajectories. The blue line shows the quadratic trend. (B) Model-predicted episodic memory change over time since baseline, stratified by bilingualism. Because the follow-up interval was substantially shorter than the full chronological age range shown in the top panel, longitudinal change was modeled and plotted as approximately linear, consistent with the primary mixed-effects model. The x-axis indicates time since baseline (dA); the y-axis shows *z*-scored episodic memory adjusted for baseline age (A0), ethnicity, education, gender, and practice. Solid-colored lines represent fitted trajectories for monolingual and bilingual groups.

The mixed-effects model on episodic memory revealed the same A0 × dA interaction, χ^2^(1) = 22.14, *p* < .001, *η*^2^*p <* .001 (Figure 2A), but in this case, the effect of aging (dA) also interacted with bilingualism, χ^2^(1) = 7.03, *p* = .03, *η*^2^*p =* .003: bilingual participants showed a slower decline in memory compared with monolinguals (Figure 2B). Indeed, monolinguals showed a significant negative slope, *b* = −0.097, 95% CI [−0.18, −0.01], whereas the slope for bilinguals was not significant, *b *= 0.056, 95% CI [−0.10, 0.21]. No other effects of bilingualism reached significance (Table S17).

## Discussion

Bilingualism has long been proposed as a contributor to cognitive reserve, yet evidence remains mixed regarding its protective effects against age-related cognitive decline (Brini et al., 2020; Grasso et al., 2023). Using the large, multi-ethnic HABS-HD cohort, we found that bilingualism was associated with slower episodic memory decline within participants, regardless of ethnicity, gender, and education. This supports bilingualism as a type of cognitive reserve.

The longitudinal association between bilingualism and episodic memory aligns with established trajectories of cognitive aging (Salthouse, 2019). Episodic memory is one of the earliest cognitive functions to decline in healthy aging and is particularly sensitive to Alzheimer’s-related pathology within medial temporal lobe structures (Hedden & Gabrieli, 2004). Given this vulnerability, daily engagement in memory-intensive processes, such as managing multiple languages, may strengthen encoding and retrieval through enriched semantic associations and cross-linguistic mappings (Schroeder & Marian, 2012). Executive function, which relies more on frontoparietal networks, did not show such a bilingual advantage. This null longitudinal finding should be interpreted cautiously, particularly given prior evidence linking bilingualism to prefrontal and executive functioning (Bialystok, 2021). Executive function and episodic memory may differ in their sensitivity to longitudinal change, practice effects, and bilingualism-related influences within the present age range and follow-up period. Thus, bilingualism-related effects on executive function may become more apparent with an older baseline age, over a longer follow-up interval, or later in aging and disease progression (Park & Reuter-Lorenz, 2009). Thus, bilingualism appears to enhance cognitive reserve most clearly in those cognitive domains most vulnerable to early aging and Alzheimer’s pathology. However, the effects of bilingualism did not survive correction for the cross-sectional or longitudinal probability of a dementia diagnosis. Thus, it may be that bilingualism protects against the memory decline that is typical of healthy aging, but not necessarily that caused by Alzheimer pathology.

The association between bilingualism and a slower rate of memory decline is notable in contrast to the effect of education. Many cross-sectional studies consistently report that higher levels of education are correlated with better memory performance and reduced incidence of dementia. However, longitudinal studies, including a recent mega-analysis (Fjell et al., 2025), have shown that education elevates baseline memory but does not affect the subsequent rate of memory decline (nor the rate of brain atrophy). This pattern may reflect early-life or genetic factors that influence both cognitive ability and educational attainment, rather than a protective effect of education in later life. Consequently, individuals with lower baseline memory may appear more susceptible to dementia on cross-sectional cognitive tests, even if their decline rate is typical. In contrast, the significantly slower rate of memory decline in bilinguals in the present study suggests that bilingualism, unlike education, may be truly protective to some extent.

At the same time, cross-sectional analyses showed that the effects of bilingualism on baseline cognitive performance differed by ethnicity, with Hispanic bilinguals showing advantages in both episodic memory and executive function, while Black bilinguals showed no reliable benefit or even a potential disadvantage in episodic memory. Rather than reflecting cognitive reserve, we suspect these between-participant differences are confounded with other sociocultural differences between bilinguals and monolinguals. This novel demonstration that the effects of bilingualism can depend on factors such as ethnicity is important because it may help explain some of the discrepancies between previous cross-sectional studies, which have tested different ethnicities and/or different sociocultural contexts.

One possible explanation for why the effect of bilingualism varied with ethnicity is that it reflects a true consequence of differential language experience. The SASH scores (Table 1) reveal distinct language-use profiles across groups: the Hispanic group was significantly more likely to engage in daily language switching between Spanish and English compared to the Black and White groups, who predominantly reported using English. Frequent language switching may provide sustained practice with cognitively demanding operations, which also benefits performance on cognitive tests. By contrast, the White bilinguals are often elective second-language learners, whose lower demands for daily language switching likely diminish any cognitive benefits. The same may be true for the Black group, though the effect of bilingualism in this group should be interpreted with caution given the relatively small number of bilinguals (only 33).

An alternative explanation is that the bilingual benefit in the Hispanic group reflects sociocultural differences rather than language use per se. For example, monolingual Hispanics often face greater residential deprivation and prevalence of illiteracy (Tee et al., 2024) and may experience higher levels of daily stress, which is likely to reduce cognitive performance (non-linguistic factors that may not have been sufficiently captured by our education covariate). Of course, both linguistic and non-linguistic factors might contribute, e.g., the benefit of regularly using two languages might only emerge in populations with lower average socioeconomic resources (Bialystok, 2021); or conversely, the higher average socioeconomic resources in the White group might mask any detectable benefit of bilingual experience (Stern, 2012).

Several limitations should be acknowledged. As an observational study, causal inference is limited, and residual confounding by socioeconomic and contextual factors cannot be excluded. Although education was included as a covariate, it is only one indicator of SES and may not fully capture income, literacy, education quality, occupational complexity, healthcare access, neighborhood context, or other environmental determinants of cognitive aging. This issue is further complicated by the uneven distribution of bilingualism and ethnicity in the sample: bilingual participants were predominantly Hispanic, which reduces the power to distinguish bilingualism-related from ethnicity-related differences. In addition, the relatively young baseline age of the cohort and the limited follow-up period of up to 6 years may also have limited sensitivity to detect bilingualism-related differences in dementia prevalence and effects on cognitive measures. Bilingualism was defined as a categorical variable based on self-report and did not capture proficiency, frequency, or context of language use, which may differentially influence cognitive outcomes (Hack et al., 2019; Zahodne et al., 2014). Future studies should use continuous and multidimensional measures of bilingualism to better understand how different aspects of the bilingual experience relate to cognitive aging. Finally, effect sizes for certain outcomes were modest, suggesting that the clinical magnitude of the associations should be interpreted with caution.

Despite these limitations, this study provides some of the first longitudinal evidence from a large, ethnically diverse cohort that bilingualism may protect against age-related declines in cognition, specifically episodic memory. The results reinforce the difficulties of interpreting effects of bilingualism in the presence of moderators such as ethnicity; moderators that themselves may reflect sociocultural differences between various communities. For geriatric practice, lifelong language history should be considered during cognitive assessment, as bilingual experience may influence both baseline performance and subsequent trajectories. Further longitudinal research integrating detailed language measures and biological markers is needed to clarify mechanisms and implications for dementia prevention.

## Supplementary Material

gbag150_Supplementary_Data

## Data Availability

The HABS-HD data supporting the findings of this study are available to the scientific community through the University of North Texas Health Science Center Institute for Translational Research (ITR) website.
