# Supplementary material for: Bilingualism and cognitive aging in ethnically diverse older adults: cross-sectional and longitudinal evidence from Health and Aging Brain Study—Health Disparities
Source: J Gerontol B Psychol Sci Soc Sci. 2026 Jul 25;81(10):gbag150. doi: 10.1093/geronb/gbag150 (PMC13600381; doi:10.1093/geronb/gbag150)
Supplement: Supplementary file 1 [file gbag150_supplementary_data.pdf]

The Journals of Gerontology, Series B: Psychological Sciences and Social Sciences
Supplementary Material: Xia et al. Bilingualism and Cognitive Aging in Ethnically Diverse Older Adults: Cross-sectional and Longitudinal Evidence from HABS-HD

Contents

1 Baseline (Cross-sectional) Analysis 2
1.1 Dementia Status as Outcome 2
1.2 Cognitive Performance 3
1.2.1 EFA of Baseline Cognitive Performance 4
1.2.2 Executive Function 5
1.2.3 Episodic Memory 6
1.3 Language Performance 7
1.3.1 English 7
1.3.2 Spanish 9
1.4 Subset analysis focusing on bilinguals with completed L2 history 10
2 Longitudinal Analysis 11
2.1 Overall monolingual and bilingual sample characteristics 11
2.2 EFA of cognitive performance in longitudinal sub-sample 12
2.3 Cognitive Stability in Initially Cognitively Unimpaired Participants 13
2.4 Cognitive Performance 14
2.4.1 Executive Function 14
2.4.2 Episodic Memory 15

1 Baseline (Cross-sectional) Analysis

1.1 Dementia Status as Outcome

Table S1 Logistic Regression Predicting Probable Dementia (PD) From Bilingualism, Gender, Ethnicity, Age, and Education

**Predictor**	***LR*** χ^2	***df***	***p (raw)***	***p (Holm)***	η^2_p
Bilingualism	1.690	1	0.194	1.000	<.001
Gender	70.562	1	<.001	–	0.020
Ethnicity	93.348	2	<.001	–	0.020
poly(Age, 2)	64.993	2	<.001	–	0.020
EducationYears	32.619	1	<.001	–	<.001
Bilingualism × Gender	0.323	1	0.570	1.000	<.001
Bilingualism × Ethnicity	8.052	2	0.018	0.143	<.001
Gender × Ethnicity	2.418	2	0.299	–	<.001
Bilingualism × poly(Age, 2)	0.713	2	0.700	1.000	<.001
Gender × poly(Age, 2)	0.121	2	0.941	–	<.001
Ethnicity × poly(Age, 2)	8.494	4	0.075	0.255	<.001
Bilingualism × Gender × Ethnicity	0.182	2	0.913	1.000	<.001
Bilingualism × poly(Age, 2) × Gender	1.285	2	0.526	1.000	<.001
Bilingualism × poly(Age, 2) × Ethnicity	4.889	4	0.299	1.000	<.001
Gender × Ethnicity × poly(Age, 2)	12.348	4	0.015	–	<.001
Bilingualism × Gender × poly(Age, 2) × Ethnicity	2.257	4	0.689	1.000	<.001

Note. Values are likelihood-ratio χ^2 statistics from a logistic regression predicting dementia status (0 = cognitively unimpaired, 1 = probable dementia). Holm–Bonferroni–corrected p values are reported only for effects involving Bilingualism. Reference groups were Hispanic (ethnicity), male (gender), and monolingual (bilingualism). η^2_p = partial eta squared. Full model: glm(PD ~ Bilingualism × poly(Age, 2) × Gender × Ethnicity + EducationYears, family = binomial).
Significance: * *p* <.05, ** *p* <.01, *** *p* <.001.

Table S2 Logistic Regression Predicting Probable Dementia (PD) among Hispanic Participants

**Predictor**	***LR*** χ^2	***df***	***p***	***OR***	**95% CI**
Bilingualism	7.00	1	.008	0.84	[0.74, 0.96]
Gender	11.05	1	<.001	0.81	[0.72, 0.91]
poly(Age, 2)	35.37	2	<.001	—	—
EducationYears	1.75	1	.185	0.92	[0.82, 1.04]
Bilingualism × Gender	0.34	1	.563	1.04	[0.92, 1.17]
Bilingualism × poly(Age, 2)	2.11	2	.348	—	—
Gender × poly(Age, 2)	6.65	2	.036	—	—
Bilingualism × poly(Age, 2) × Gender	1.41	2	.495	—	—

Note. Full model: glm(PD ~ Bilingualism × poly(Age, 2) × Gender + EducationYears, family = binomial).

1.2 Cognitive Performance

To maximise sample size, PCA was performed on data concatenated across all participants and time-points. Before PCA, time-based scores were reverse-coded (i.e., their sign was reversed) to ensure that higher values meant better performance for all scores. The results of PCA analysis are shown in Figure S1. Parallel analysis on resampled data suggested that two components were sufficient, explaining 66.1% of the data variance. The exploratory factor analysis (EFA) with two factors and oblique rotation resulted in a factor that we labelled Executive Function (capturing 35.1% of the variance), since it loaded significantly on the TMT-A, TMT-B, DSST, Animal fluency, FAS, SEVLT-total, DSF, and DFD tasks, and a second factor that we labelled Episodic Memory (capturing 24.2% of the variance), since it loaded on LM I, LM II, SEVLT-total, and SEVLT-DR tasks. The precise loadings are presented in Table S3.

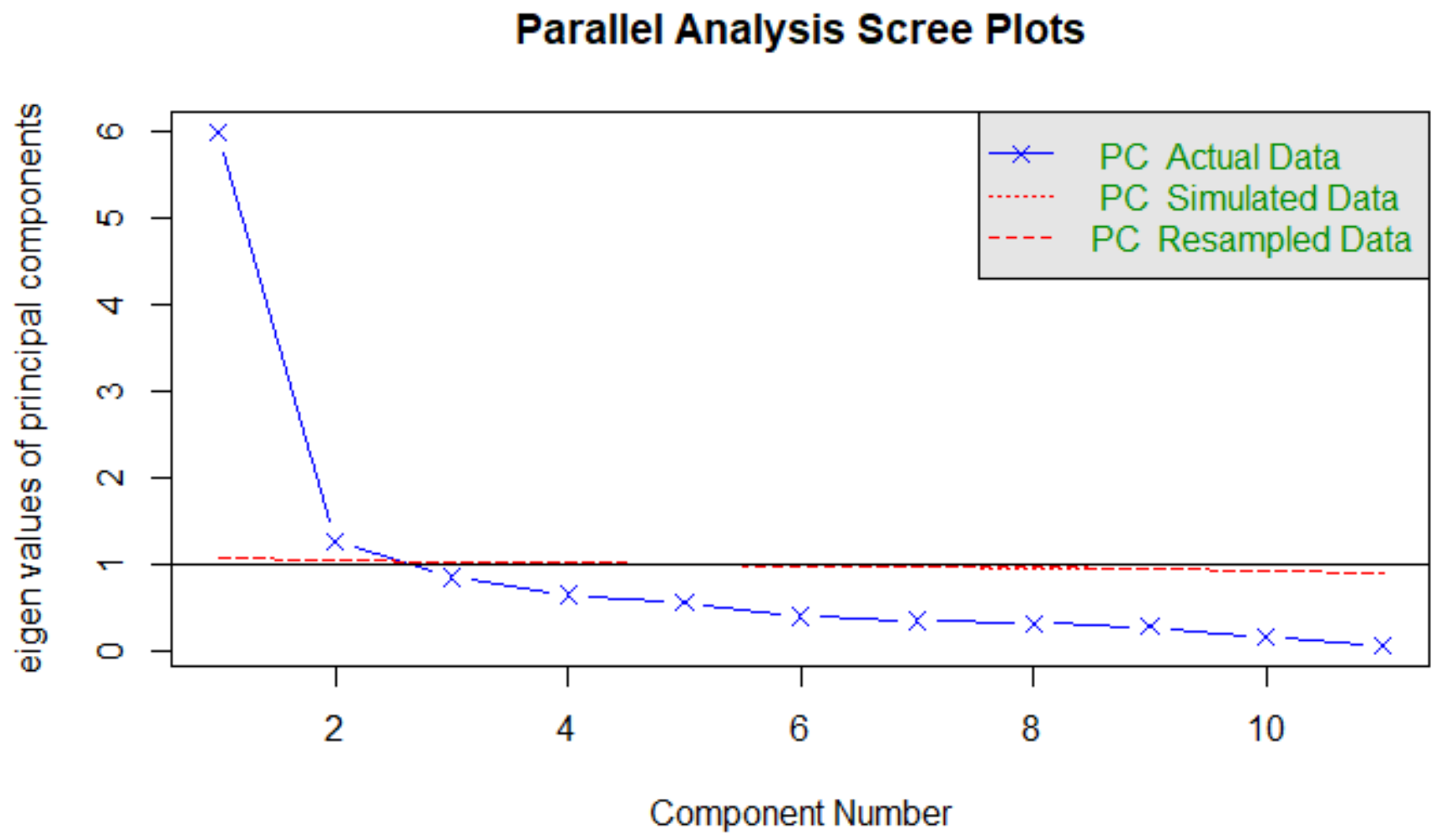

Figure S1 Parallel analysis scree plot showing eigenvalues for actual data (PCs: ×), simulated data (dotted lines), and resampled data (dashed lines). Components with eigenvalues exceeding the 95th percentile of simulated or resampled data are considered significant. The first three components surpass these thresholds and are retained.

Table S3 Standardized Factor Loadings for the Three-Factor Exploratory Factor Analysis

Variable	Factor 1 (Executive Function)	Factor 2 (Episodic Memory)	Communality
Trails A – Reversed	.82*	—	.60
Trails B – Reversed	.81*	—	.69
Digit Symbol Substitution	.87*	—	.72
Digit Span Forward	.57*	—	.32
Digit Span Backward	.56*	.31	.41
Logical Memory I	—	.93*	.88
Logical Memory II	—	1.00*	.94
SEVLT Immediate Recall	.31*	.51*	.56
SEVLT Delayed Recall	—	.48*	.50
FAS Verbal Fluency	.64*	—	.47
Animal Naming	.54*	—	.44

Note. *p* <.01. Loadings <.30 are suppressed.

1.2.1 EFA of Baseline Cognitive Performance

To confirm that the two-factor structure was not specific to the above pooled approach, the factor analyses were repeated within a longitudinal structural equation modelling framework (ESEM), even though this entailed a reduced sample size (*n* = 184 with 4 Visits). A two-factor solution fitted significantly better than a one-factor solution, and the loading pattern at each wave closely matched the pooled solution in Table S3. The standardised loadings from the four-wave configural solution are presented in Table S4.

Table S4 Standardised Factor Loadings for the Four-Wave Configural Longitudinal ESEM

Variable	Visit 1		Visit 2		Visit 3		Visit 4	
	EF	EM	EF	EM	EF	EM	EF	EM
Trails A – Reversed	.67*	—	.61*	—	.70*	—	.73*	—
Trails B – Reversed	.68*	—	.66*	—	.73*	—	.85*	—
Digit Symbol Substitution	.85*	—	.91*	—	.84*	—	.94*	—
Digit Span Forward	.65*	—	.58*	—	.57*	—	.63*	—
Digit Span Backward	.61*	—	.67*	—	.59*	—	.65*	—
Logical Memory I	—	.78*	—	.77*	—	.81*	—	.88*
Logical Memory II	—	.90*	—	.93*	—	.93*	—	.99*
SEVLT Immediate Recall	.35*	.36*	.32*	.43*	.38*	.43*	.34*	.40*
SEVLT Delayed Recall	.33*	.30*	.33*	.39*	.45*	—	.38*	.36*
FAS Verbal Fluency	.45*	—	.52*	—	.55*	—	.58*	—
Animal Naming	.42*	—	.49*	—	.51*	—	.48*	—

Note. *p* <.01. Loadings <.30 are suppressed.

To test for measurement invariance across waves, a longitudinal confirmatory factor analysis was employed, wherein Episodic Memory was indicated by Logical Memory I, Logical Memory II, and SEVLT Immediate and Delayed Recall, while Executive Function was indicated by the remaining tests, together with SEVLT Immediate and Delayed Recall, which cross-loaded onto this factor. SEVLT measures were allowed to cross-load since it showed to be a better fit than when attributed uniquely to EM factor ($\Delta\chi^2$ = 89.00, *p* <.001), similar to the pooled data solution.

Constraining the loadings to equality across four visits did not significantly worsen fit relative to the configural model ($\Delta\chi^2$ = 41.58, p = .145), establishing metric measurement invariance. The two-factor structure is therefore data-driven and stable over the study period, supporting the use of a two-factor model pooled across waves to maximise the use of all available data.

1.2.2 Executive Function

Table S5 Effects of Bilingualism, Age, Education, and Ethnicity on Executive Function

Predictor	*df*	*F*	*p (raw)*	*p (Holm)*	η^2_p
Bilingualism	1, 4103	20.49	<.001***	<.001	.005
Age (poly, 2)	2, 4103	483.93	<.001***	—	.190
Gender	1, 4103	134.94	<.001***	—	.030
Ethnicity	2, 4103	165.61	<.001***	—	.070
Education years	1, 4103	1291.76	<.001***	—	.240
Bilingualism × Age	2, 4103	3.69	.025*	.151	.002
Bilingualism × Gender	1, 4103	0.30	.581	.581	<.001
Age × Gender	2, 4103	0.66	.516	—	<.001
Bilingualism × Ethnicity	2, 4103	11.74	<.001***	<.001	.006
Age × Ethnicity	4, 4103	6.02	<.001***	—	.006
Gender × Ethnicity	2, 4103	1.39	.250	—	<.001
Bilingualism × Age × Gender	2, 4103	3.14	.043*	.174	.002
Bilingualism × Age × Ethnicity	4, 4103	1.79	.127	.255	.002
Bilingualism × Gender × Ethnicity	2, 4103	3.58	.028*	.151	.002
Age × Gender × Ethnicity	4, 4103	2.17	.070	—	.002
Bilingualism × Age × Gender × Ethnicity	4, 4103	2.15	.072	.217	.002

Note. Holm–Bonferroni correction was applied only to effects involving Bilingualism**.** Reference groups: Hispanic (ethnicity), male (gender), monolingual (bilingualism). η^2_p = partial eta squared. Full model: Executive_Function ~ Bilingualism × poly(Age, 2) × Gender × Ethnicity + EducationYears
Significance levels: † *p* <.10, * *p* <.05, ** *p* <.01, *** *p* <.001.

Table S6 Two-Way Interaction of Bilingualism and Ethnicity on Executive Function: Ethnicity-Specific Breakdown

Ethnicity	Predictor	*F*	*df*	*p*	*Std. β*	95% CI
**Hispanic**	Bilingualism	30.24	1	<.001	0.10	[0.07, 0.14]
	Gender	25.84	1	<.001	0.09	[0.06, 0.13]
	Age (poly1)	143.54	2	<.001	-11.29	[-12.62, -9.96]
	Age (poly2)	143.54	2	<.001	-2.18	[-3.48, -0.87]
	EducationYears	921.03	1	<.001	0.59	[0.55, 0.62]
**Black**	Bilingualism	3.39	1	.066	-0.13	[-0.27, 0.01]
	Gender	52.43	1	<.001	0.17	[0.12, 0.22]
	Age (poly1)	181.15	2	<.001	-14.45	[-15.99, -12.90]
	Age (poly2)	181.15	2	<.001	-3.97	[-5.52, -2.42]
	EducationYears	304.53	1	<.001	0.40	[0.35, 0.44]
**White**	Bilingualism	0.22	1	.639	0.02	[-0.06, 0.09]
	Gender	50.80	1	<.001	0.17	[0.12, 0.21]
	Age (poly1)	156.47	2	<.001	-14.80	[-16.49, -13.10]
	Age (poly2)	156.47	2	<.001	-3.76	[-5.45, -2.06]
	EducationYears	153.33	1	<.001	0.29	[0.24, 0.33]

Note. The full model for example: Executive_Function ~ Bilingualism * Gender + poly(Age,2) + EducationYears

1.2.3 Episodic Memory

Table S7 Effects of Bilingualism, Age, Ethnicity, and Education on Episodic Memory

Predictor	*df*	*F*	*p (raw)*	*p (Holm)*	η^2_p
Bilingualism	1, 4103	3.06	.080†	.563	<.001
Age (poly, 2)	2, 4103	125.30	<.001***	—	.060
Gender	1, 4103	156.09	<.001***	—	.040
Ethnicity	2, 4103	169.07	<.001***	—	.080
Education years	1, 4103	502.11	<.001***	—	.110
Bilingualism × Age	2, 4103	0.12	.885	1.000	<.001
Bilingualism × Gender	1, 4103	0.93	.336	1.000	<.001
Age × Gender	2, 4103	1.23	.292	—	<.001
Bilingualism × Ethnicity	2, 4103	5.61	.004**	.030	.003
Age × Ethnicity	4, 4103	1.60	.171	—	.002
Gender × Ethnicity	2, 4103	0.88	.416	—	<.001
Bilingualism × Age × Gender	2, 4103	2.01	.135	.701	<.001
Bilingualism × Age × Ethnicity	4, 4103	1.11	.350	1.000	<.001
Bilingualism × Gender × Ethnicity	2, 4103	2.15	.117	.701	<.001
Age × Gender × Ethnicity	4, 4103	0.84	.500	—	<.001
Bilingualism × Age × Gender × Ethnicity	4, 4103	1.57	.178	.713	.002

Note. Holm–Bonferroni correction was applied only to effects involving Bilingualism. Age was modeled as a second-order polynomial. Reference groups: Hispanic (ethnicity), male (gender), monolingual (bilingualism). η^2_p = partial eta squared. Full model: Episodic_Memory ~ Bilingualism × poly(Age, 2) × Gender × Ethnicity + EducationYears
Significance levels: * *p* <.05, ** *p* <.01, *** *p* <.001.

Table S8 Two-Way Interaction of Bilingualism and Ethnicity on Episodic Memory: Ethnicity-Specific Breakdown

Ethnicity	Predictor	*F*	*df*	*p*	*Std. β*	95% CI
**Hispanic**	Bilingualism	19.26	1	<.001	0.11	[0.06, 0.16]
	Gender	29.29	2	<.001	-5.03	[-6.77, -3.29]
	Age (poly1)	29.29	2	<.001	-4.47	[-6.17, -2.76]
	Age (poly2)	33.52	1	<.001	0.14	[0.09, 0.18]
	EducationYears	226.64	1	<.001	0.38	[0.33, 0.43]
**Black**	Bilingualism	5.56	1	.019	-0.18	[-0.34, -0.03]
	Gender	45.52	2	<.001	-7.23	[-8.95, -5.52]
	Age (poly1)	45.52	2	<.001	-4.16	[-5.88, -2.44]
	Age (poly2)	44.20	1	<.001	0.17	[0.12, 0.23]
	EducationYears	221.49	1	<.001	0.38	[0.33, 0.42]
**White**	Bilingualism	0.18	1	.673	-0.02	[-0.10, 0.06]
	Gender	54.47	2	<.001	-8.65	[-10.46, -6.85]
	Age (poly1)	54.47	2	<.001	-4.12	[-5.92, -2.31]
	Age (poly2)	71.12	1	<.001	0.21	[0.16, 0.26]
	EducationYears	107.04	1	<.001	0.26	[0.21, 0.31]

Note. The full model for example: Episodic_Memory~ Bilingualism * Gender + poly(Age,2) + EducationYears

1.3 Language Performance

To examine whether bilingualism was associated with differences in L1 performance, separate models were fit for English and Spanish. English performance (AMNART, reversed z-scored so that higher scores mean better performance) was compared between English monolinguals and English-dominant bilinguals. The same predictors were used as above. Spanish performance (WAT, z-scored) was examined in Hispanic participants only, comparing monolingual-Spanish speakers and bilingual-Spanish speakers. The same predictors were used except without the Ethnicity factor. Thus, participants were categorized as: Monolingual English (ME), Monolingual Spanish (MS), Bilingual English-first language (BE), Bilingual Spanish-first language (BS), or Bilingual Other-first language (BO). This category was only for the analysis of first language performance.

1.3.1 English

Age showed a small negative association on the test of English language proficiency, *F* (2, 2971) = 9.71, p <.001, η^2_p <.001, with older age linked to slightly lower AMNART scores. Gender was positively associated, *F* (1, 2971) = 22.44, p <.001, η^2_p <.001, with females scoring higher than males. Education was positively related to AMNART performance, *F* (1, 2971) = 1010.39, p <.001, η^2_p = .25, with more years of education associated with better scores. The main effect of Ethnicity was significant, *F* (2, 2971) = 472.75, p <.001, η^2_p = .24: with White > Hispanic > Black (see Table S9).

The effect of Bilingualism on English language proficiency interacted with Ethnicity, *F* (2, 2971) = 6.21, p = .002, η^2_p = .004. Follow-up analyses by Ethnicity revealed that Hispanic participants exhibited a bilingual disadvantage, *F* (1, 481) = 5.76, p = .017, (β = -0.11, 95% *CI* [-0.21, -0.02]), with bilingual-English speakers performing worse (more errors) than monolingual-English speakers. There was no significant bilingualism effect among Black participants, *F* (1, 1163) = 3.30, p = .070, η^2_p <.001. Conversely, White participants demonstrated a bilingual advantage, *F* (1, 1346) = 8.78, p = .003, (β = 0.15, 95% *CI* [0.05, 0.25]), with bilingual-English speakers outperforming monolingual-English speakers (see Figure S2, Table S10).

Table S9 Effects of Bilingualism, Age, Gender, Ethnicity, and Education on English Performance (AMNART)

**Predictor**	***df***	***F***	***p (raw)***	***p (Holm)***	η^2_p
Bilingualism	1, 2971	0.34	.562	1.000	<.001
Gender	1, 2971	22.44	<.001***	—	.008
Ethnicity	2, 2971	472.75	<.001***	—	.240
Age (poly, 2)	2, 2971	9.71	<.001***	—	.006
Education years	1, 2971	1010.39	<.001***	—	.250
Bilingualism × Gender	1, 2971	1.11	.293	1.000	<.001
Bilingualism × Ethnicity	2, 2971	6.21	.002**	.016	.004
Gender × Ethnicity	2, 2971	0.02	.983	—	<.001
Bilingualism × Age	2, 2971	1.71	.181	1.000	.001
Gender × Age	2, 2971	3.56	.029*	—	.002
Ethnicity × Age	4, 2971	1.91	.107	—	.003
Bilingualism × Gender × Ethnicity	2, 2971	0.53	.588	1.000	<.001
Bilingualism × Gender × Age	2, 2971	0.88	.414	1.000	<.001
Bilingualism × Ethnicity × Age	4, 2971	1.32	.258	1.000	.002
Gender × Ethnicity × Age	4, 2971	0.73	.568	—	<.001
Bilingualism × Gender × Ethnicity × Age	4, 2971	1.81	.124	.867	.002

Note. Reference groups: Hispanic (ethnicity), male (gender), monolingual (bilingualism). η^2_p = partial eta squared.
Full model: AMNART ~ Bilingualism × poly(Age, 2) × Gender × Ethnicity + EducationYears

Table S10 Two-Way Interaction of Bilingualism and Ethnicity on English Performance: Ethnicity-Specific Breakdown

**Ethnicity**	**Predictor**	*F*	*df*	*p*	*Std. β*	95% CI
**Hispanic**	Bilingualism	5.76	1	.017	−0.11	[−0.21, −0.02]
	Gender	0.75	2	.473	−0.86	[−2.48, 0.77]
	Age (poly1)	0.75	2	.473	0.53	[−1.07, 2.12]
	Age (poly2)	0.86	1	.353	0.04	[−0.04, 0.11]
	EducationYears	241.39	1	<.001 ***	0.58	[0.51, 0.65]
**Black**	Bilingualism	3.30	1	.070	0.18	[−0.01, 0.36]
	Gender	7.76	2	<.001 ***	−1.92	[−3.59, −0.25]
	Age (poly1)	7.76	2	<.001 ***	−2.76	[−4.44, −1.08]
	Age (poly2)	4.34	1	.037	0.05	[0.00, 0.11]
	EducationYears	404.65	1	<.001 ***	0.51	[0.46, 0.55]
**White**	Bilingualism	8.78	1	.003 **	0.15	[0.05, 0.25]
	Gender	6.51	2	.002 **	−1.23	[−2.96, 0.50]
	Age (poly1)	6.51	2	.002 **	−2.93	[−4.66, −1.20]
	Age (poly2)	17.75	1	<.001 ***	0.10	[0.05, 0.15]
	EducationYears	356.31	1	<.001 ***	0.46	[0.41, 0.51]

Note. The full model for example: AMNART ~ Bilingualism + poly(Age,2) + Gender + EducationYears

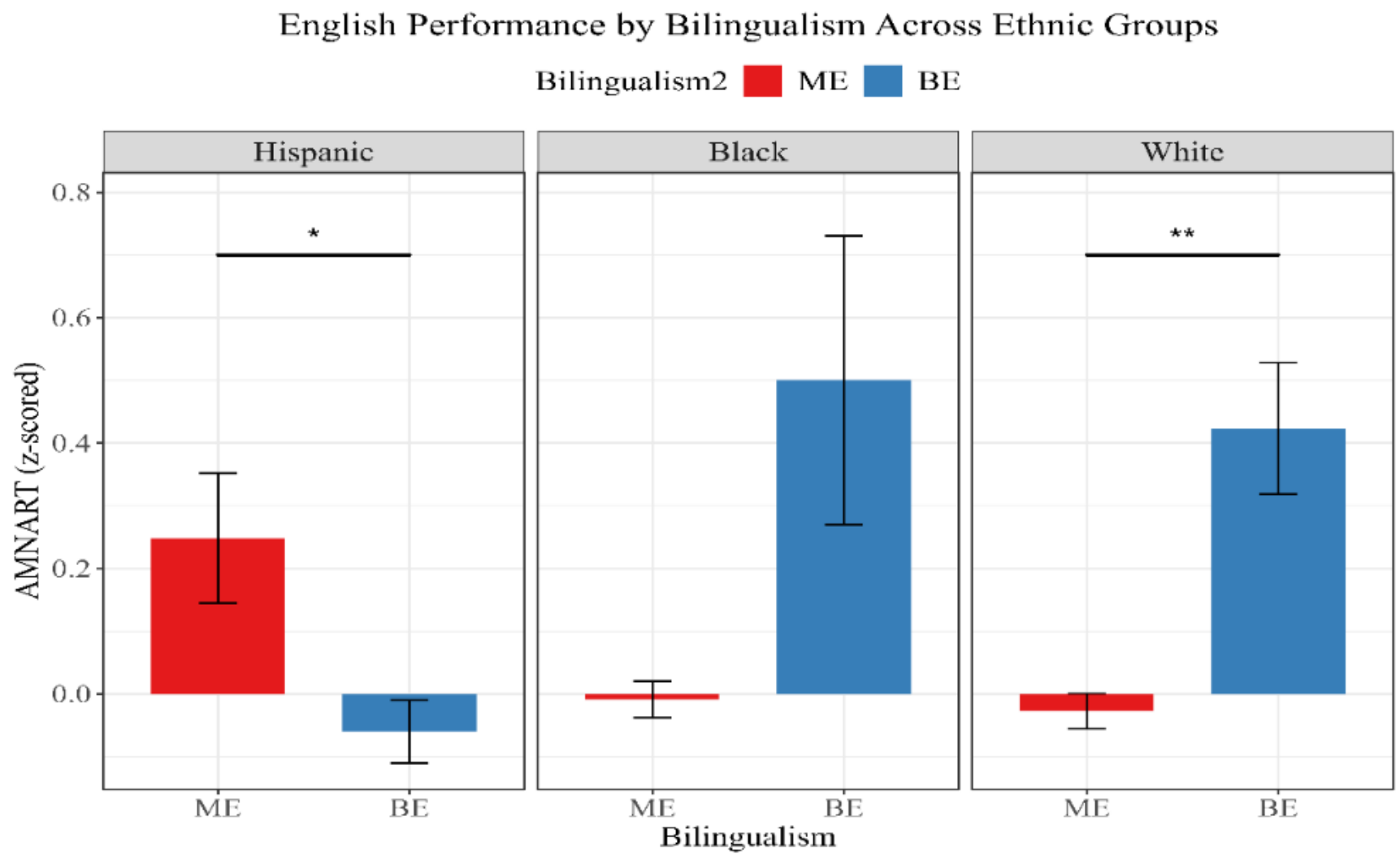

Figure S2 English Performance Z-scores by bilingualism across ethnic groups. Error bars represent ±1 SE. Asterisks indicate significant group differences, based on the same statistical tests reported in the main text (** *p* <0.01; * *p* <0.05). ME = Monolingual English; BE = Bilingual English.

1.3.2 Spanish

Performance on the test of Spanish language proficiency in the Hispanic participants revealed no significant effects of Bilingualism, Age, or Gender. Education was the only significant positive predictor, F (1, 860) = 404.49, p <.001, η^2_p = .32, with more years of education associated with better scores (see Table S11).

Table S11 Effects of Bilingualism, Age, Gender, and Education on Spanish Performance

**Predictor**	***df***	***F***	***p***	η^2_p
Bilingualism	1, 860	2.14	.144	.002
Gender	1, 860	0.98	.323	.001
Age (poly, 2)	2, 860	1.71	.182	.004
Education years	1, 860	404.49	<.001***	.320
Bilingualism × Gender	1, 860	0.13	.716	<.001
Bilingualism × Age	2, 860	0.74	.476	.002
Gender × Age	2, 860	2.75	.064†	.006
Bilingualism × Gender × Age	2, 860	1.55	.214	.004

Note. The full model: WAT ~ Bilingualism2 * poly(Age,2) * Gender + EducationYears

1.4 Subset analysis focusing on bilinguals with completed L2 history

Analyses examining the contributions of L2 history to cognitive performance in bilinguals revealed a consistent pattern across executive function (EF) and episodic memory (EM). Higher L2 proficiency was significantly associated with higher EF, F (1, 81) = 11.88, p = .001 (β = 0.49, 95% CI [0.21, 0.77]), and better EM, F (1, 81) = 8.98, p = .004, (β = 0.45, 95% CI [0.15, 0.75]), such that individuals with greater proficiency in their second language demonstrated stronger cognitive abilities. In contrast, L2 age of acquisition (AoA), L2 usage, and frequency of code-switching did not significantly predict either EF or EM (all p >.05). These findings suggest that, among the various indices of bilingual experience, L2 proficiency is the primary factor supporting cognitive function, while other L2 history variables show no reliable effect (see Table S12). This subset analysis was restricted to participants with complete L2 history data.

Table S12 Effects of L2 History on EF and EM performance

Predictor	*F (df)*	*p*	*Std. β*	95% CI
**Executive Function**				
Gender	0.44 (1,81)	.510	0.06	[-0.13, 0.25]
Age [poly,1st]	6.22 (2,81)	.003	-2.94	[-4.64, -1.24]
Age [poly,2nd]			-0.90	[-2.68, 0.88]
EducationYears	8.81 (1,81)	.004	0.29	[0.10, 0.49]
L2 Proficiency	11.88 (1,81)	.001	0.49	[0.21, 0.77]
L2 AoA	0.37 (1,81)	.547	0.06	[-0.14, 0.26]
L2 Usage	1.72 (1,81)	.194	-0.17	[-0.44, 0.09]
CodeSwitch	0.12 (1,81)	.730	0.03	[-0.16, 0.23]
**Episodic Memory**				
Gender	2.69 (1,81)	.105	0.17	[-0.04, 0.37]
Age [poly,1st]	1.93 (2,81)	.151	-1.64	[-3.47, 0.18]
Age [poly,2nd]			-0.87	[-2.78, 1.03]
EducationYears	4.45 (1,81)	.038	0.22	[0.01, 0.43]
L2 Proficiency	8.98 (1,81)	.004	0.45	[0.15, 0.75]
L2 AoA	1.80 (1,81)	.183	0.15	[-0.07, 0.36]
L2 Usage	2.20 (1,81)	.142	-0.21	[-0.49, 0.07]
CodeSwitch	0.21 (1,81)	.651	0.05	[-0.16, 0.26]

Note: L2 Proficiency**:** 1 = Almost None; 2 = Very Poor; 3 = Fair; 4 = Functional; 5 = Good; 6 = Very Good; 7 = Like Native Speaker; L2 Usage**:** 0 = Never; 1 = Rarely; 2 = Sometimes; 3 = Mostly; 4 = Almost Always; Code-Switching: 0 = Never; 1 = Very Infrequently; 2 = Occasionally; 3 = Two or Three Times per Conversation; 4 = Several Times per Conversation; 5 = Constantly.

Full model: Executive_Function ~ Gender + poly(Age,2) + EducationYears + L2_Proficiency + L2AoA + L2Usage + CodeSwitch; Episodic_Memory ~ Gender + poly(Age,2) + EducationYears + L2_Proficiency + L2AoA + L2Usage + CodeSwitch

2 Longitudinal Analysis

2.1 Overall monolingual and bilingual sample characteristics

Bilingual participants were younger than monolinguals (p <.001). Education was lower among bilinguals (p <.001). MMSE total scores were slightly lower in bilinguals (M = 27.99, SD = 2.63) than monolinguals (M = 27.72, SD = 2.07), p <.001. Gender distribution did not differ between groups (p = .54). Ethnic composition varied significantly: the majority of bilinguals were Hispanic (81.2%), whereas monolinguals were predominantly White (55%) or Black (26.2%), p <.001. Additionally, bilingual participants had fewer follow-up visits on average, with a higher proportion completing only two visits (53.3% vs. 68.9% for monolinguals; see Table S13).

Table S13 Participant Demographic Information across Language Groups (Longitudinal Sample)

Variable	Hispanic		White		Black	
	Monolingual (n = 232)	Bilingual (n = 424)	Monolingual (n = 681)	Bilingual (n = 83)	Monolingual (n = 325)	Bilingual (n = 15)
A0, M (SD)	63.35 (7.91)	63.33 (7.64)	68.58 (8.22)	68.00 (8.32)	62.96 (7.59)	62.62 (5.23)
dA, M (SD)	1.91 (1.96)	2.07 (2.06)	2.00 (2.00)	2.07 (2.07)	1.10 (1.13)	1.11 (1.16)
Gender, %						
Male	57 (24.6)	141 (33.3)	278 (40.8)	39 (47.0)	102 (31.4)	13 (86.7)
Female	175 (75.4)	283 (66.7)	403 (59.2)	44 (53.0)	223 (68.6)	2 (13.3)
Education (years), M (SD)	7.94 (4.36)	11.70 (3.82)	15.69 (2.39)	16.31 (2.53)	14.86 (2.59)	15.60 (2.16)
MMSE Total, M (SD)	25.41 (3.54)	27.48 (2.05)	28.94 (1.64)	28.95 (1.83)	27.85 (2.27)	27.73 (1.49)
PD conversion %						
PD	38 (21.1)	76 (22.7)	62 (10.7)	7 (9.5)	24 (11.7)	0 (0.0)
Max Visit ID, %						
2 visits	143 (61.6)	218 (51.4)	388 (57.0)	45 (54.2)	323 (99.4)	15 (100.0)
3 visits	65 (28.0)	140 (33.0)	210 (30.8)	27 (32.5)	2 (0.6)	0 (0.0)
4 visits	24 (10.3)	66 (15.6)	83 (12.2)	11 (13.3)	0 (0.0)	0 (0.0)

Note. Values are presented as Mean (SD) for continuous variables and n (%) for categorical variables. A0 = baseline age; dA = time since baseline (years); MMSE_Total = Mini-Mental State Examination total score; Group = Dementia Status (PD = probable dementia; Stable CU = stable cognitively unimpaired); Max_Visit_ID = highest number of visits completed.

2.2 EFA of cognitive performance in longitudinal sub-sample

As with the full baseline sample, a two-factor EFA with oblimin rotation was conducted, resulting in CFI = .87 and RMSEA = .18, with the two factors accounting for 58.1% of the total variance (Factor 1 = 34.5%, Factor 2 = 23.6%). The factor loadings were very similar to those for the full baseline sample (as expected) and are presented in Table S14.

Table S14 Standardized Factor Loadings for the Three-Factor Exploratory Factor Analysis

**Variable**	**Factor 1 (Executive Function)**	**Factor 2 (Episodic Memory)**	**Communality**
Trails A – Reversed	0.784*	—	0.560
Trails B – Reversed	0.800*	—	0.672
Digit Symbol Substitution	0.872*	—	0.709
Digit Span Forward	0.579*	—	0.325
Digit Span Backward	0.556*	—	0.396
Logical Memory I	—	0.913*	0.874
Logical Memory II	—	1.011*	0.957
SEVLT Immediate Recall	0.342*	0.468*	0.541
SEVLT Delayed Recall	—	0.483*	0.497
FAS Verbal Fluency	0.628*	—	0.456
Animal Naming	0.540*	—	0.404

Note. *p* <.01. Loadings <.30 are suppressed.

2.3 Cognitive Stability in Initially Cognitively Unimpaired Participants

Table S15 Logistic Regression Predicting Probability of PD Conversion in CU Participants

**Predictor**	***df***	***LR*** χ^2	***p* (raw)**	***p* (Holm)**	η^2_p
Bilingualism	1	0.20	.656	1.000	<.001
Age (poly, 2)	2	45.99	<.001***	—	.040
Gender	1	17.65	<.001***	—	.010
Ethnicity	1	14.87	<.001***	—	.010
Education years	1	3.99	.046*	—	.003
Bilingualism × Age	2	0.17	.919	1.000	<.001
Bilingualism × Gender	1	1.96	.162	.648	.002
Bilingualism × Ethnicity	1	0.23	.634	1.000	<.001

Note. PD = probable dementia; CU = cognitively unimpaired.
Full model: PD ~ Bilingualism * (poly(A0,2) + Gender + Ethnicity) + EducationYears

2.4 Cognitive Performance

Because the primary longitudinal question concerned cognitive change over time, interpretation focused on terms involving Bilingualism × dA.

2.4.1 Executive Function

Table S16 Longitudinal Effects of Bilingualism, Gender, Age, Education, and Ethnicity on Executive Function

**Predictor**	χ^2	***df***	***p (raw)***	***p (Holm)***	η^2_p
A0	409.32	1	<.001	—	.190
dA	28.45	1	<.001	—	.010
Bilingualism	10.61	1	0.001	—	.006
Gender	57.98	1	<.001	—	.030
Ethnicity	185.81	2	<.001	—	.090
EducationYears	467.13	1	<.001	—	.190
Practice	36.29	3	<.001	—	.010
A0 × dA	106.00	1	<.001	—	.040
A0 × Bilingualism	0.57	1	0.450	—	<.001
dA × Bilingualism	0.66	1	0.418	0.967	<.001
A0 × Gender	0.52	1	0.469	—	<.001
dA × Gender	1.41	1	0.235	—	<.001
Bilingualism × Gender	1.45	1	0.229	—	<.001
A0 × Ethnicity	8.14	2	0.017	—	.005
dA × Ethnicity	5.47	2	0.065	—	.002
Bilingualism × Ethnicity	10.88	2	0.004	—	.006
Gender × Ethnicity	0.52	2	0.771	—	<.001
A0 × dA × Bilingualism	0.09	1	0.771	—	<.001
A0 × dA × Gender	1.94	1	0.163	—	<.001
A0 × Bilingualism × Gender	0.37	1	0.545	—	<.001
dA × Bilingualism × Gender	0.02	1	0.896	0.967	<.001
A0 × dA × Ethnicity	2.78	2	0.249	—	.001
A0 × Bilingualism × Ethnicity	3.96	2	0.138	—	.002
dA × Bilingualism × Ethnicity	4.07	2	0.131	0.523	.002
A0 × Gender × Ethnicity	1.17	2	0.556	—	<.001
dA × Gender × Ethnicity	0.07	2	0.966	—	<.001
Bilingualism × Gender × Ethnicity	1.51	2	0.470	—	<.001
A0 × dA × Bilingualism × Gender	0.11	1	0.735	—	<.001
A0 × dA × Bilingualism × Ethnicity	9.19	2	0.010	—	.004
A0 × dA × Gender × Ethnicity	3.96	2	0.138	—	.002
A0 × Bilingualism × Gender × Ethnicity	10.72	2	0.005	—	.006
dA × Bilingualism × Gender × Ethnicity	2.26	2	0.322	0.967	<.001
A0 × dA × Bilingualism × Gender × Ethnicity	1.06	2	0.589	—	<.001

Note. χ^2 = Wald chi-square statistic; *df* = degrees of freedom; *p* (raw) = uncorrected p-value; *p* (Holm) = Holm–Bonferroni corrected p-value for effects involving Bilingualism; η^2_p = partial eta-squared, representing effect size. Holm–Bonferroni correction was applied only to effects that include key interactions of interest involving dA and Bilingualism, that are dA*Bilingualism, dA*Bilingualism*Gender, dA*Bilingualism*Ethnicity, and dA*Bilingualism*Gender*Ethnicity. Reference groups: Hispanic (ethnicity), male (gender), monolingual (bilingualism).
Full linear mixed-effects mode: Executive_Function ~ A0 * dA * Bilingualism * Gender * Ethnicity + EducationYears + Practice + (1|Med_ID).
Significance codes: * *p* <.05, ** *p* <.01, *** *p* <.001.

2.4.2 Episodic Memory

Table S17 Longitudinal Effects of Bilingualism, Gender, Age, Education, and Ethnicity on Episodic Memory

Predictor	χ^2	*df*	*p (raw)*	*p (Holm)*	η^2_p
A0	96.72	1	<.001	—	.050
dA	6.06	1	0.014	—	.002
Bilingualism	0.63	1	0.427	—	<.001
Gender	72.16	1	<.001	—	.040
Ethnicity	196.33	2	<.001	—	.100
EducationYears	181.76	1	<.001	—	.090
Practice	38.62	3	<.001	—	.010
A0 × dA	22.14	1	<.001	—	.008
A0 × Bilingualism	0.39	1	0.534	—	<.001
dA × Bilingualism	7.03	1	0.008	0.032	.003
A0 × Gender	0.70	1	0.402	—	<.001
dA × Gender	0.05	1	0.831	—	<.001
Bilingualism × Gender	3.03	1	0.082	—	.002
A0 × Ethnicity	2.99	2	0.224	—	.002
dA × Ethnicity	3.28	2	0.194	—	.001
Bilingualism × Ethnicity	2.74	2	0.254	—	.002
Gender × Ethnicity	0.32	2	0.854	—	<.001
A0 × dA × Bilingualism	0.91	1	0.341	—	<.001
A0 × dA × Gender	0.55	1	0.460	—	<.001
A0 × Bilingualism × Gender	0.41	1	0.521	—	<.001
dA × Bilingualism × Gender	0.00	1	0.960	1.000	<.001
A0 × dA × Ethnicity	2.97	2	0.227	—	.001
A0 × Bilingualism × Ethnicity	3.27	2	0.195	—	.002
dA × Bilingualism × Ethnicity	2.70	2	0.260	0.780	.001
A0 × Gender × Ethnicity	1.45	2	0.485	—	<.001
dA × Gender × Ethnicity	0.25	2	0.881	—	<.001
Bilingualism × Gender × Ethnicity	0.25	2	0.884	—	<.001
A0 × dA × Bilingualism × Gender	1.86	1	0.173	—	<.001
A0 × dA × Bilingualism × Ethnicity	4.00	2	0.136	—	.002
A0 × dA × Gender × Ethnicity	4.43	2	0.109	—	.002
A0 × Bilingualism × Gender × Ethnicity	2.13	2	0.345	—	.001
dA × Bilingualism × Gender × Ethnicity	0.49	2	0.784	1.000	<.001
A0 × dA × Bilingualism × Gender × Ethnicity	0.82	2	0.662	—	<.001

Note. χ^2 = Wald chi-square statistic; *df* = degrees of freedom; *p* (raw) = uncorrected p-value; *p* (Holm) = Holm–Bonferroni corrected p-value for effects involving Bilingualism; η^2_p = partial eta-squared, representing effect size. Holm–Bonferroni correction was applied only to effects that include key interactions of interest involving dA and Bilingualism, that are dA*Bilingualism, dA*Bilingualism*Gender, dA*Bilingualism*Ethnicity, and dA*Bilingualism*Gender*Ethnicity. Reference groups: Hispanic (ethnicity), male (gender), monolingual (bilingualism). Full linear mixed-effects mode: Episodic_Memory ~ A0 * dA * Bilingualism * Gender * Ethnicity + EducationYears + Practice + (1|Med_ID).
Significance codes: * *p* <.05, ** *p* <.01, *** *p* <.001.
